# Protective Effects of SIRT6 Overexpression against DSS-Induced Colitis in Mice

**DOI:** 10.3390/cells9061513

**Published:** 2020-06-22

**Authors:** Kang Xu, Yannan Guo, Lu Ping, Ying Qiu, Qingfei Liu, Zhongchi Li, Zhao Wang

**Affiliations:** 1 Protein Science Key Laboratory of the Ministry of Education, School of Pharmaceutical Sciences, Tsinghua University, Beijing 100084, China; xukang16@mails.tsinghua.edu.cn (K.X.); gyn19@mails.tsinghua.edu.cn (Y.G.); liuqf@tsinghua.edu.cn (Q.L.); 2 8-year MD Program, Chinese Academy of Medical Sciences and Peking Union Medical College, Beijing 100730, China; lu_ping@student.pumc.edu.cn; 3 School of Medicine, Tsinghua University, Beijing 100084, China; yingqiu2009@tsinghua.edu.cn

**Keywords:** SIRT6, colitis, inflammation, NF-κB and c-Jun signaling, TAK1

## Abstract

Sirtuin 6 (SIRT6), as a NAD + -dependent deacetylase, plays an indispensable role in the regulation of health and physiology. Loss of SIRT6 causes spontaneous colitis in mice and makes intestinal epithelial cells prone to stress. However, whether SIRT6 overexpression increases resistance to colitis remains unknown. Here, in vivo results demonstrated that SIRT6 overexpression attenuates DSS-induced colitis in terms of clinical manifestations, histopathological damage, loss of tight junction function and imbalanced intestinal microenvironment. Additionally, we also found that the activation of NF-κB and c-Jun induced by DSS is diminished by SIRT6 overexpression. Furthermore, SIRT6 may regulate TAK1 to inhibit NF-κB and c-Jun signaling. Thus, our findings highlight the protective effect of SIRT6 on colon, further supporting the perspective that SIRT6 may be a therapeutic target for intestine injury under stress.

## 1. Introduction

SIRT6, as an adenosine diphosphate (ADP)-ribosyl transferase and NAD + -dependent deacetylase, has been associated with metabolism, longevity regulation, and many other essential biological processes [1]. It has been reported that SIRT6 is involved in vascular inflammation, as knockdown of SIRT6 increases the proinflammatory cytokines production in human umbilical vein endothelial cells (HUVECs) [2]. SIRT6-deficient mice experience severe cellular and physiological inflammation, including type 1 interferon response, LINE 1 activation, and sterile inflammation [3]. In parallel, overexpressed SIRT6 is thought to ameliorate a variety of age-related disorders. Our previous studies showed that SIRT6 overexpression attenuates cisplatin-induced acute kidney injury, including inflammation and apoptosis, by inhibiting ERK1/2 signaling [4]. FY Liu reported that intestinal epithelial cell-specific knockout of SIRT6 increases susceptibility to dextran sulfate sodium salt (DSS)-induced colitis in mice. Moreover, there is a decreased expression of colonic SIRT6 in both DSS-induced colitis mouse model and ulcerative colitis patients [5]. However, the effects of overexpressed SIRT6 on the colon have not yet been elucidated.

Inflammatory bowel diseases (IBD), clinically including Crohn’s disease (CD) and ulcerative colitis (UC), are characterized by chronic inflammation of the gastrointestinal tract [6,7]. A variety of factors contribute to the morbidity of IBD, consisting of the interaction of genetic, diet, microbial, molecular, immunological, environmental and drug use-related factors [6]. DSS-induced experimental colitis is most commonly used to understand the IBD pathogenesis [7,8]. Disruption of the epithelial barrier in DSS-induced colitis results in the increased inflammatory cell infiltration and excessive production of pro-inflammatory cytokines, causing destructive effects [9]. Our previous study discovered that SIRT6 overexpression protects kidney epithelial cells from cisplatin-induced inflammation and apoptosis, which has led us to wonder if it can protect the intestine as well.

To investigate whether SIRT6 overexpression holds protective effects against DSS-induced colitis, we examined the weight loss, colitis symptoms, histological tissue damage, expression of tight junction-associated proteins, and production of pro-inflammatory cytokines [10]. Our results demonstrated that SIRT6 overexpression significantly attenuates DSS-induced clinical manifestations, colonic histological damage and intestinal dysfunction in mice. Notably, overexpressed SIRT6 efficiently suppressed the excessive activation of both NF-κB and c-Jun induced by DSS. The phosphorylation of TAK1, an upstream signaling molecule of NF-κB and c-Jun, was altered by DSS, and also was regulated by SIRT6. Taken together, our results reveal the pivotal protective effects of SIRT6 against DSS-induced colitis, mainly through inhibiting the activation of NF-κB and c-Jun by regulating TAK1 signaling. Our study further supports the conclusion that SIRT6 is indispensable for the maintenance of health and provides a new therapeutic target for colitis.

## 2. Materials and Methods

### 2.1. Animals

SIRT6 Transgenesis mice were generated from the C57BL6 strain. Supported by Cyagen Transgenic Animal Center (Suzhou, China), cDNA amplified from a plasmid encoding mouse SIRT6 was subcloned to the cytomegalovirus promoter vector with an enhanced green fluorescent protein. Then the transgenesis vector was microinjected to mice pronuclei to obtain SIRT6 overexpression mice embryo. Transgene positive mice selection were carried out at the age of 1 to 2 weeks. Their tail DNA were obtained and genotyped with the following PCR primers: forward, 5′-GCCGTCTGGTCATTGTCAACCTG-3′; reverse, 5′-AAAGACCCCTAGGAATGCTCGTCAA-3′. The transgene positive strain had 494 bp PCR production. After over four generations of crossbreeding, we selected the stable and SIRT6 high expression line for further research. All animals were maintained under specific pathogen-free (SPF) animal facilities with 12 h light and 12 h dark cycle with 22 ± 1 °C temperature and 55 ± 5% of relative humidity. Mice were fed with standard AIN-93G diet and water. All the animal procedures complied with regulations and ethics guidelines which were approved by the International Animal Care and Use Committee (IACUC) of Tsinghua University (18-WZ1, 26 March 2018).

### 2.2. Colitis Induction

To avoid potential impact, colitis-induced mice and their littermates were co-housed and acclimatized for over a week. Colitis was induced in mice following the protocol published before [11]. Briefly, mice (male, 10–12 weeks old) fed a normal diet were administered 3% dextran sulfate sodium salt (DSS) (MW 36–50 kDa; MP Biomedical, Irvine, CA, USA) dissolved in drinking water for 7 days. Control littermates were given regular drinking water. During induction, mice were weighed and stools were tested for occult blood daily to assess pathology severity. At the end of induction time points, mice were euthanized by CO_2_ inhalation. Colon and other tissue were collected and snap frozen in liquid nitrogen and store at −80 °C for further use. Blood was centrifugated in 1000 g for 30 min at 4 °C to isolate serum. The serum was stored at −80 °C for further use as well.

### 2.3. Pathology Scoring

The course of inflammation was monitored by weight loss measurements and stools morphology test. Pathological scoring was used to quantify the severity of colitis following a method adapted from the standard DAI scoring system [12]. Each animal was examined once a day and received a score containing three parameters, body weight decreasing, fecal bleeding and stool consistency. For the assessment score, the definition was as follows: the decrease in body weight relative to initial weight reached 0–1% scored 0, 1–5% scored 1, 5–10% scored 2, 10–15% scored 3 and over 15% scored 4; the stool normal and no blood scored 0, the dimly visible streak of blood scored 1, moderate blood scored 2, obvious blood scored 3 and gross bleeding from the anus scored 4. As for the stool viscosity, normal, the score of 0; soft, 1; loss, 2; diarrhea, 3 and diarrhea with blood, 4. Then these features were averaged for each mouse and each group to calculate the DAI.

### 2.4. Immunohistochemistry Staining Assay

Immunohistochemistry was performed following the protocol described previously [13,14]. Briefly, acetone fixed frozen sections were cut from the colonic samples at a thickness of around 5 μm. Endogenous peroxidase activity was inhibited by 3% hydrogen peroxide. Antigen retrieval was performed by autoclaving at 120 °C for 15 min in 0.01 M citrate buffer at around pH 6.0. Sections then were blocked in 5% rat serum and incubated overnight at 4 °C with the diluted biotinylated primary antibody (1:500; rat-anti-mouse; anti-TAK1, #ab109526, Abcam, Cambridge, England, UK; anti-p-TAK1, #4508, Cell Signaling Technology, Danvers, MA, USA; anti-c-Jun, #ab40766, Abcam; anti-p-Jun, #ab32385, Abcam; anti-NF-κB p65 antibody, #ab32536, Abcam; anti-NF-κB p65 (phosphor S536), #ab86299, Abcam; anti-NF-κB p65 (acetyl K310) antibody, #ab19870, Abcam). Sections were washed three times and then DAPI (1:1000, Abcam) was used to contrast staining. Fluorescence images were acquired using a Zeiss LSM510 (Jena, Germany) confocal microscope. We used ImageJ version 1.48 (Wayne Rasband, National Institutes of Health, Bethesda, MD, USA) under Java 1.6.0_20 (64-bit, ORACLE, Redwood City, CA, USA) environment to analyze the positive staining areas of immunohistochemistry images. We used ImageJ plugin: Immunohistochemistry (IHC) Image Analysis Toolbox downloaded from the website: https://imagej.nih.gov/ij/plugins/. We strictly followed the ImageJ plugin IHC Image Analysis Toolbox protocol to evaluate immunostained slides [15]. We evaluated the IHC-positively stained cells and/or area ratio to interpret and present the IHC data referring the evaluation method described by Mashimo et al. [16]. We randomly and blindly measured the staining intensity of each slice with the help of ImageJ software. For those slides which IHC positively staining area were congregated in cell nucleus, we analyzed the proportion of immunohistochemically stained positive cells flexibly. For those slices with relatively intensive background staining, to avoid misinterpretation, we used the color threshold tool in ImageJ to filter the positively stained area with RGB value. Finally, results were calculated on average according to different experiment groups and displayed in bar plot.

### 2.5. Histological Staining

Samples of colon tissue were fixed in 4% paraformaldehyde for a day at 4 °C and then embedded in paraffin and stained with hematoxylin and eosin after dehydrated. To detect the glycogen in tissue, formalin-fixed, paraffin-embedded tissue sections underwent PAS staining. The procedures were carried out as follows: Tissue was first deparaffinized and hydrated to water. Then 0.5% periodic acid solution was used to oxidization for 5 min. After rinsing in distilled water, the tissue was placed in Schiff reagent for 15 min until the section became light pink color. The tissue section was then washed in lukewarm tap water for 5 min and the section turned dark pink color. Mayer’s hematoxylin was used to counterstain for a minute and the tissue was washed in tap water for 5 min. Finally, dehydration and slip covering were used by a synthetic mounting medium. To stain acid mucocutaneous and acetic mucins, some of the colonic tissue were used for Alcian Blue staining. Briefly, slides were deparaffinized and hydrated to distilled water and stained in Alcian Blue solution for 30 min. Then they were washed under running tap water for 2 min and rinsed in distilled water. After being counterstained in a nuclear fast red solution for 5 min, they were washed in running tap water for 1 min. The slides were then dehydrated through 95% alcohol, 2 changes of absolute alcohol for 3 min each. Finally, the slides were cleared in xylene or xylene substitute and mounted with resinous mounting medium. To calculate the goblet cells ratio of each slice, we randomly chose five calculation fields from each Alcian Blue staining slice. Calculated the number of goblet cells and the number of other unstained cells and gained the ratio of goblet cells in each calculation field. For every Alcian Blue staining slice, we calculated the average of five calculation fields as the outcome and summarized the final result into the bar plot.

### 2.6. Histological and Macroscopic Assessing

With H&E, PAS and Alcian Blue stained slides, we measured three independent parameters to assess the histological scores. The first parameter is the severity of inflammation, with a score ranging from 0 to 3, indicating no inflammation, mild, moderate, or severe inflammation. The second parameter is mucosal damage, with a score ranging from 0 to 3, representing none, mucosa, mucosa, or submucosa, transmembrane. The third parameter is crypt damage, with a score of 0 to 4, representing none, one third of basal are damaged, basal two-thirds are damaged, only the epithelium is intact, and the entire crypt and epithelium are lost. Then, we estimated the percentage of tissue involvement. Each parameter scored was multiplied by the percentage of tissue involved and the total was added up to obtain the histopathological score. The observation was aided by a light microscope (Leica, Wetzlar, Germany) and software Zen 2.3 (blue edition, Carl Zeiss Microscopy GmbH, 2011) in a blinded manner. Mice were subsequently sacrificed using carbon dioxide. The appearances of intraperitoneal intestine were taken before the further autopsy. The colonic length and morphology were used to assess colitis severity.

### 2.7. Cytokine and MPO Measurements

The tissue samples from the colon were homogenized with phosphate buffer saline and centrifuged for 15 min at 8000 rpm at 4 °C. The supernatant was collected for cytokines and MPO measurements. The measurement of cytokines and MPO from serum and colonic supernatant were made by ELISA kit (Cloud Clone Corp., Wuhan, China). The assay was performed according to the manufacturer’s instructions. A total volume of 50 μL diluted (1:2.5) sample was used in each well. The assay was run in duplicate for each sample. A GLOMAX Multi Detection System (Promega, Madison, WI, USA) was used for the plate reader analysis. Raw data from standard curves and analyzed through MSD Discovery Workbench software (version 4.0, MESO SCALE DIAGNOSTICS, Rockville, MD, USA).

### 2.8. Quantitative Real-Time Reverse Transcription PCR Assay

Total RNA was isolated from freeze-thawed colon tissue using Trizol (Thermo Fisher Scientific, Waltham, MA, USA). RNA concentrations and purity were estimated by determining the A260/A230 ratio with a Thermo Scientific Nanodrop 2000c (Thermo Scientific, Waltham, MA, USA). Reverse transcription of mRNA was performed using the cDNA Synthesis Kit (TIANGEN, Beijing, China). PCR was carried out using SYBR Green with CFX Manager 3.1 (Bio-Rad, Hercules, California, USA). Each sample was processed in triplicate and normalized to GAPDH by the 2-ΔΔCT method, and the values were expressed relative to those of the control group. The following primers were used: SIRT6 (forward: CAGTACGTCAGAGACACGGTTG; reverse: GTCCAGAATGGTGTCTCTCAGC). GAPDH (forward: CATCACTGCCACCCAGAAGACTG; reverse: ATGCCAGTGAGCTTCCCGTTCAG). occludin (forward: ATGGCAAAGTGAATGACAAGCGG; reverse: CTGTAACGAGGCTGCCTGAAGT). ZO-1 (forward: GTCCAGAATCTCGGAAAAGTGCC; reverse: CTTTCAGCGCACCATACCAACC).

### 2.9. Western Blot Analysis

Total proteins from the colon tissues were extracted in RIPA lysis buffer (Solarbio, Beijing, China), supplemented with a cocktail of protease (Solarbio) and phosphatase inhibitors (Solarbio), and then diluted with 6 × loading buffer to the same concentration and denatured. Protein samples were run on 12% SDS-PAGE and electrotransferred to polyvinylidene difluoride membranes (Millipore, Bedford, MA, USA). After blocking with 5% skim milk containing 0.1% fetal bovine serum (Gibco, Waltham, MA, USA) for 45 min at 37 °C, the membranes were incubated with primary antibody diluent for 12 h at 4 °C. The blots were then incubated with corresponding horseradish peroxidase (HRP)-conjugated secondary antibody (Abcam) for 2 h. The protein bands were visualized using enhanced chemiluminescence reagent (Santa, Dallas, TX, USA) and a Chemi Capture (CLINX, Shanghai, China). Primary antibodies against the proteins were as follows: SIRT6 (#ab191385, Abcam), GAPDH (#ab181602, Abcam).

### 2.10. Statistical Analysis

Results are expressed as means ± sem. Statistical significance was evaluated using ANOVA and a post-Tukey-Kramer test. A *p*-value of less than 0.05 was considered significant. Data were analyzed and plotted in Graph Pad Prism 6.0 software.

## 3. Results

### 3.1. SIRT6 Overexpression Attenuated DSS-Induced Clinical Manifestations of Colitis in Mice

**Figure 1:**
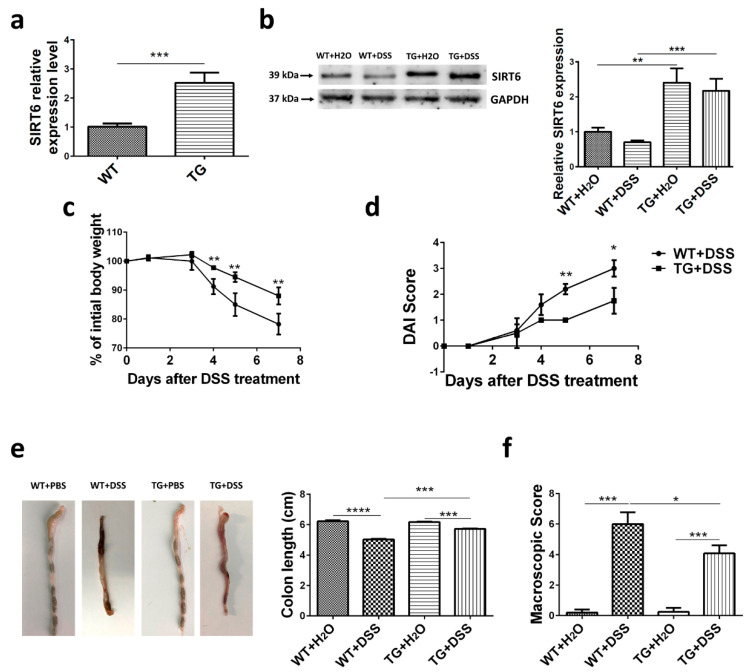
Clinical manifestations induced by DSS were attenuated in SIRT6 transgenic mice. WT and SIRT6 TG mice were supplied with regular drinking water and drinking water containing 3% DSS for 7 days. (**a**) mRNA levels of colonic SIRT6 in WT and SIRT6 TG mice. (**b**) Protein levels of colonic SIRT6 in mice. (**c**) Weight change within 7 days of DSS treatment. (**d**) DAI score within 7 days of DSS treatment. (**e**) Colon appearance and length on day 7. (**f**) Macroscopic score at day 7. *n* = 6–8, * *p* < 0.05, ** *p* < 0.01, *** *p* < 0.001, **** *p* < 0.0001.

We previously generated a SIRT6 transgenic mouse model [4]. QPCR and Western blot analysis of total colon tissue protein showed that SIRT6 was overexpressed in TG groups (Figure 1a,b). Western blot also revealed a slightly but not significantly decreased expression of SIRT6 after 7 days of DSS treatment in WT mice, but not in TG mice (Figure 1b). To study the protective capability of SIRT6, we induced a DSS colitis model in mice on day 1 according to the established protocol, with only minor modifications (see Method). We tested various clinical manifestations in different groups, including wild type mice with H_2_O (WT + H_2_O) or DSS (WT + DSS), SIRT6 transgenic mice with H_2_O (TG + H_2_O) or DSS (TG + DSS). The induction of colitis with DSS caused a significant loss of body weight after 7-day administration, and the weight loss began on day 3 of DSS treatment. However, SIRT6 overexpression significantly attenuated the loss of body weight after DSS treatment (Figure 1c). The DAI scores of mice in DSS treatment groups increase apparently over time and TG + DSS mice manifested a mild decrease in DAI scores compared with WT + DSS mice (Figure 1d). Furthermore, DSS treatment resulted in a reduction of colon length which was attenuated by SIRT6 overexpression (Figure 1e). Based on the situation of stool bleeding, we analyzed the macroscopic score and, notably, SIRT6 significantly attenuated DSS-induced stool bleeding (Figure 1f). Collectively, these results demonstrated that SIRT6 overexpressed mice showed increased tolerance to DSS-induced colitis and maintained a more normal bowel function.

### 3.2. SIRT6 Overexpression Improved Histological Injury Induced by DSS in Mice

**Figure 2:**
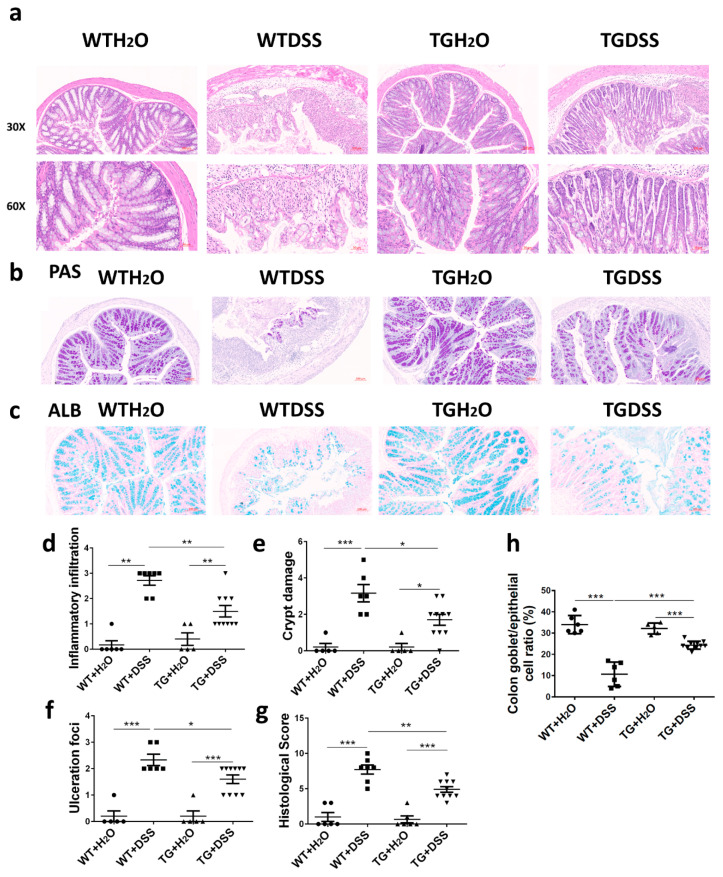
SIRT6 overexpression attenuated the histopathological damage induced by DSS in mice. (**a**–**c**) Representative images of histopathology of sections of colon tissue that were prepared from water-treated WT and SIRT6 mice and DSS-treated WT and SIRT6 TG mice and stained with H&E, PAS or ALB. (**d**–**g**) Inflammatory infiltration, crypt damage, ulceration foci and the histological score of the different groups of mice. (**h**) Colon goblet cell percentage of the different groups of mice. *n* = 5–10, * *p* < 0.05, ** *p* < 0.01, *** *p* < 0.001.

Epithelial destruction, inflammatory infiltration, and crypt damage are key indicators of colon tissue injury. H&E-stained tissue samples displayed an apparent alleviation of inflammatory infiltration and crypt damage in TG + DSS group compared with WT + DSS group (Figure 2a,d,e). In addition, Periodic Acid-Schiff (PAS) staining and Alcian Blue (ALB) staining exhibited prominent goblet cell loss and decreased mucus secretion in WT + DSS mice while TG + DSS mice showed less severe (Figure 2b,c). The histological score, including the evaluation of immune cell infiltration (Figure 2d), crypt distortion (Figure 2e), and ulceration foci (Figure 2f), were all significantly higher in WT + DSS mice than those in TG + DSS mice (Figure 2g). TG + DSS mice also exhibited more percentage of colon goblet cells (Figure 2h). Taken together, these data indicated that SIRT6 overexpression preserved the integrity of the epithelial barrier and alleviated the histological damage caused by DSS.

### 3.3. SIRT6 Improved the Cellular Permeability and Integrity under the Condition of DSS Treatment

**Figure 3:**
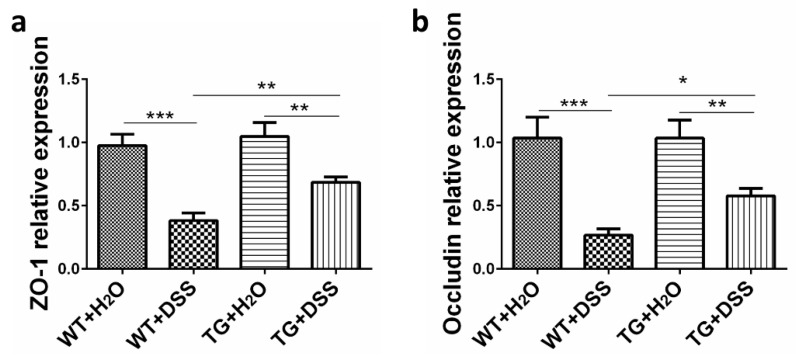
Modulation of the expression of ZO-1 and occludin. qPCR was used to detect ZO-1 (**a**) and occludin (**b**) expression in colon tissue. GAPDH was used as a control. *n* = 4, * *p* < 0.05, ** *p* < 0.01, *** *p* < 0.001.

Epithelial destruction is further measured by assessing the expression of tight junction (TJ) associated proteins, including zonula occludens-1 (ZO-1) and occludin, which prevent harmful substances from passing the mucosal epithelial cell interspaces, ensure cellular permeability and integrity, and maintain intestinal microenvironment homeostasis [17,18]. As shown in Figure 3, the expression of ZO-1 and occludin was distinctly decreased in WT + DSS mice compared to the control group. However, the levels of both ZO-1 and occludin were enhanced in TG + DSS mice compared with WT + DSS mice (Figure 3). These results suggest that SIRT6 maintains the integrity of the epithelial barrier and intestinal microenvironment under the condition of DSS treatment.

### 3.4. SIRT6 Diminished Pro-Inflammatory Cytokine Production and MPO Activity in the Colon and Serum

**Figure 4:**
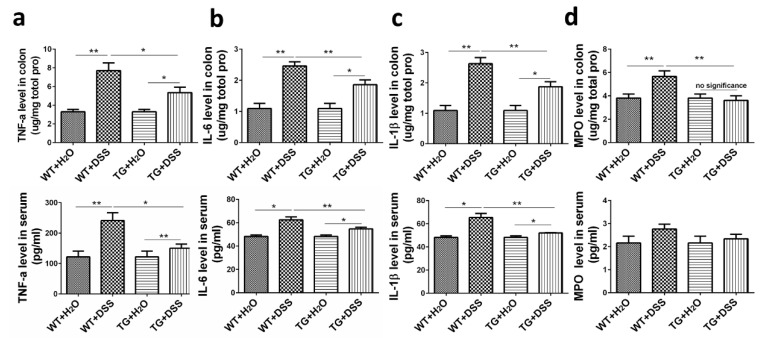
SIRT6 regulated pro-inflammatory cytokines and MPO level in colon tissue and serum of DSS-induced colitis mice. (**a**–**c**) The concentration of the pro-inflammatory cytokines including TNFα, IL6, IL-1β in the supernatant of colon tissue and serum was measured by ELISA (upper: colon tissue; lower: serum). (**d**) The MPO level in the supernatant of colon tissue and serum were measure by ELISA (upper: colon tissue; lower: serum). *n* = 6–8, * *p* < 0.05, ** *p* < 0.01.

Excessive secretion of pro-inflammatory cytokines is closely related to exacerbated intestinal inflammation [7]. We therefore explored whether SIRT6 overexpression influenced the level of pro-inflammatory cytokines in DSS-induced colitis. For this purpose, we measured the concentration of TNFα, IL6, and IL-1β in colon tissue and serum. As shown in Figure 4, the increased concentration of all these cytokines was detected in colon tissue and serum in DSS-treated mice. However, SIRT6 overexpression significantly attenuated the elevated cytokines induced by DSS (Figure 4a–c). Furthermore, we sought to investigate the activity of myeloperoxidase (MPO), an enzyme that reflects the degree of damage and inflammation in colon tissue [18]. Our results indicated that MPO activity in WT + DSS mice is dramatically increased compared with the control group, whereas no such effect of DSS was seen in the TG group. Under DSS treatment, SIRT6 overexpression resulted in a dramatic suppression of MPO activity compared with WT, especially in colon tissue (Figure 4d). These data suggest the protective effect of SIRT6 on colon function might result from attenuated inflammation.

### 3.5. SIRT6 Overexpression Suppressed the Activation of NF-κB in Colon Tissue

**Figure 5:**
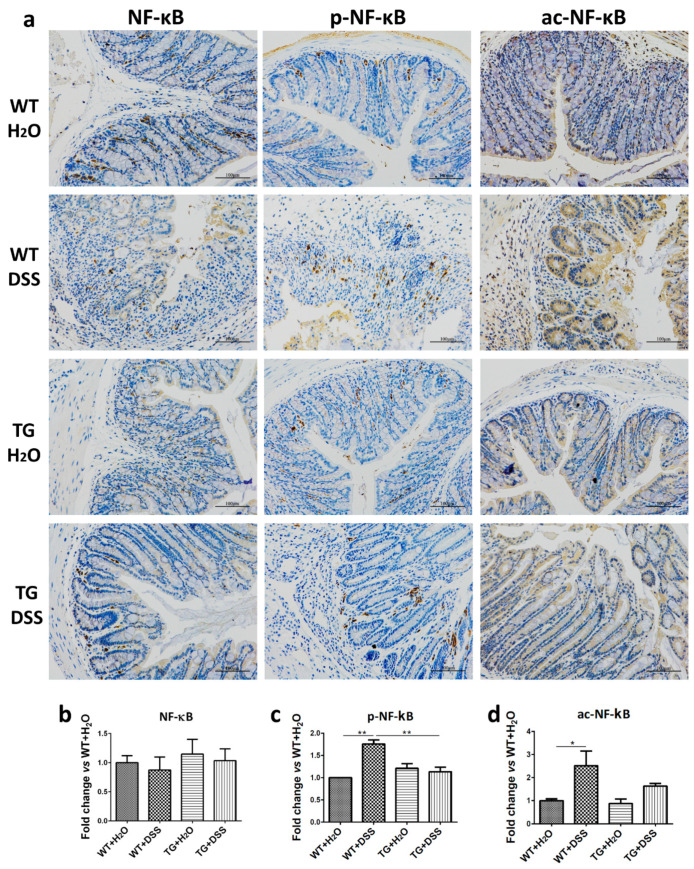
SIRT6 regulated phosphorylation and acetylation of NF-kB. (**a**) The expression, phosphorylation level, and acetylation level of NF-kB were determined by immunohistochemistry. (**b**–**d**) The statistical results of (**a**). *n* = 3–6, * *p* < 0.05, ** *p* < 0.01.

It has been reported that NF-κB plays an important role in the development of ulcerative colitis (UC) and Crohn’s disease (CD) [19]. To elevate the protective mechanism of SIRT6, we examined the phosphorylation and acetylation of NF-κB p65. Immunohistochemical results revealed that both the phosphorylation and acetylation of NF-κB p65 are obviously increased in WT + DSS mice compared with WT + H_2_O mice, but slightly increased in TG + DSS mice compared with TG + H_2_O mice (Figure 5). Thus, suppressing NF-κB activation by overexpressed SIRT6 contributes to anti-colitis in mice.

### 3.6. Inhibition of the Activation of c-Jun by SIRT6 Was Found in Colon Tissue

**Figure 6:**
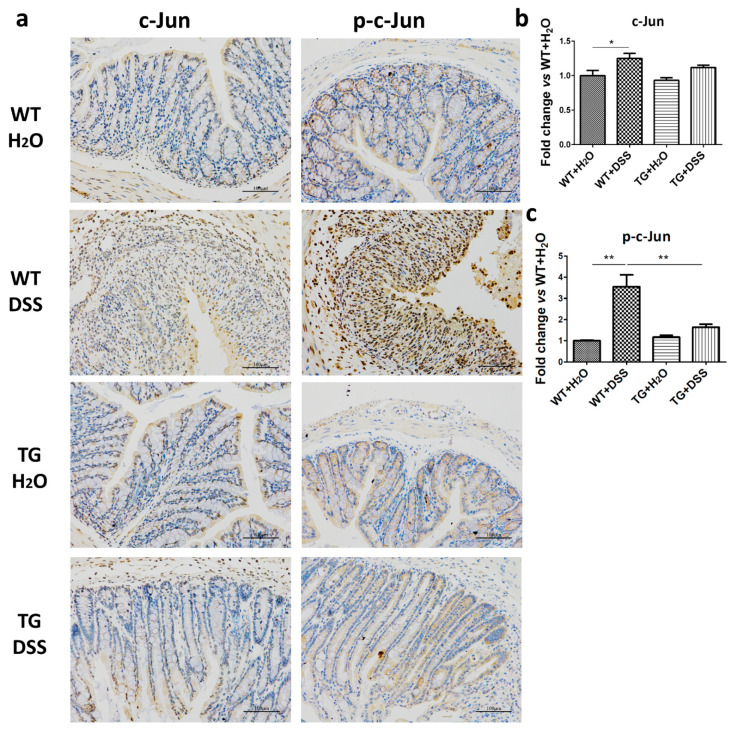
SIRT6 regulated phosphorylation of c-Jun. (**a**) The expression and phosphorylation levels of c-Jun were determined by immunohistochemistry. (**b**,**c**) The statistical results of (**a**). *n* = 3–6, * *p* < 0.05, ** *p* < 0.01.

c-Jun is a pivotal transcription factor that upregulates genes involved in immune and pro-inflammatory responses [20]. To further test the anti-inflammatory mechanisms of SIRT6, we also investigated the activation of c-Jun. In the present study, the phosphorylation of c-Jun was apparently enhanced in WT + DSS mice. However, the alteration was remarkably downregulated in TG + DSS mice compared with WT + DSS mice (Figure 6). These results indicate that c-Jun inhibition regulated by SIRT6 is involved in the anti-inflammatory action.

### 3.7. SIRT6 Overexpression Reduced the Phosphorylation of TAK1

**Figure 7:**
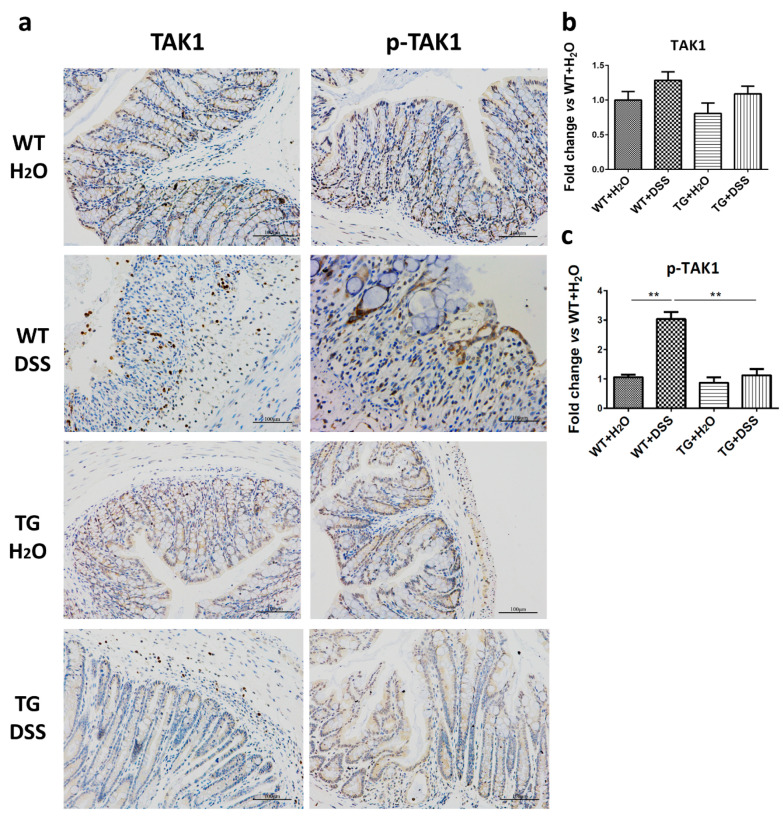
SIRT6 regulated expression and phosphorylation of TAK1. (**a**) The expression and phosphorylation levels of TAK1 were determined by immunohistochemistry. (**b**,**c**) The statistical results of (**a**). *n* = 3–6, ** *p* < 0.01.

Transforming growth factor-β activating kinase-1 (TAK1) has been reported to be an essential intermediate in several innate immune signaling pathways [21]. Additionally, TAK1 is also indispensable for the activation of both NF-κB and c-Jun, which results in increased production of cytokines and chemokines [22]. We discovered that the expression (slightly but not significantly increase) and phosphorylation of TAK1 increased by DSS treatment, and SIRT6 overexpression remarkably decreased the expression and phosphorylation level of TAK1 (Figure 7).

## 4. Discussion

SIRT6 is involved in multiple essential biological processes, including genomic stability, inflammation and metabolism, and SIRT6 deficiency, leading to severe colitis in mice and increasing susceptibility to injurious insults in cells, and its overexpression enhances resistance to stress [5,23]. However, whether SIRT6 overexpression prevents colitis has not been elucidated. In this study, we revealed that SIRT6 overexpression attenuates clinical manifestations, histological injury, loss of tight junction function, and imbalance of intestinal microenvironment in the context of DSS treatment. These results strongly suggest that SIRT6 plays a protective role against colitis.

DSS-induced colitis starts at repeated cycles of injury and healing in the intestinal epithelium [24]. We found that SIRT6 overexpression efficiently maintains the integrity of the intestinal epithelial barrier, alleviating the tissue damage caused by DSS. Goblet cells keep the inner mucus layer nutritious and robust by means continuous secretion, while PAS and ALB staining indicated that SIRT6 plays an important role in maintaining the integrity and secretory functions of goblet cells [25]. Moreover, the tight junction proteins, whose expressions were suppressed by DSS treatment, seal the paracellular space, functioning as barrier and fencing in the colon [26]. The tight junction-associated proteins, ZO-1 and occludin, were elevated in SRT6 TG mice compared to WT mice after DSS treatment. These results suggest that the attenuated colitis is correlated with the maintained expression of tight junction-associated proteins. As a key marker for acute inflammation, MPO reflects neutrophil activity biochemically and its upregulated activity is directly related to the severity of inflammation [27]. In our study, a significant increase in MPO activity was observed in WT + DSS mice, but was only a slightly elevated in TG + DSS mice.

Inhibition of NF-κB and c-Jun is widely reported to be effective in decreasing colon inflammation [28]. As important transcription factors, NF-κB and c-Jun regulate the expression of different cytokines and chemokines that contribute to the migration of immune cells to the site of inflammation and facilitates the pathogenesis of human IBD [28,29]. Our study showed that SIRT6 overexpression significantly suppresses the activation of NF-κB and c-Jun, and then reduces the expression of pro-inflammatory cytokines such as TNFα, IL-6 and IL-1β. These results are consistent with the phenomenon that TG + DSS mice have a lower level of inflammatory infiltration compared with WT + DSS mice.

TAK1, as an essential intermediate in several innate immune signaling pathways, also plays an important role in maintaining intestinal homeostasis [21]. TAK1 mediates the LPS induced NF-κB activation, which increases intestinal tight junction permeability, and the TNFα-induced c-Jun activation, which critically involves in enterocyte survival [21]. TAK1-dependent activation of NF-κB and c-Jun is responsible for the development of intestinal inflammation and the phosphorylation of TAK1 is a general signal for the activation of its targets [30]. In this study, we observed a slight but not significantly increased expression and remarkably elevated phosphorylation of TAK1 following DSS treatment; nevertheless, SIRT6 overexpression inhibited the alteration. This evidence suggests that SIRT6 may regulate the phosphorylation and activation of TAK1. TGF-β signaling has been reported to be dysregulated in IBD patients, and its impairment results in spontaneous colitis in mouse model [31]. SIRT6 could bind to and repress the expression of key TGF-β genes to decrease the activation of TGF-β signaling [32]. We speculate that TAK1, as an important component of TGF-β signaling, whose activation is to be suppressed by inhibited TGF-β signaling regulated by SIRT6 [31,32]. This still requires further research to prove.

Colonic tissue is severely damaged by DSS treatment, and multiple types of cells participate in the histological and physiological changes, such as epithelial cells, macrophages, and neutrophils [33]. Interestingly, SIRT6 plays an important role in regulating these types of cells in the process of protecting the individual from damage or stress [34,35]. SIRT6 is globally overexpressed in TG mice used in this study, so we conjecture that SIRT6 functions in various cells to prevent against DSS-induced colitis in mice.

## 5. Conclusions

Our study reveals that SIRT6 overexpression attenuates DSS-induced colitis and uncovers the protective role of SIRT6 on intestinal function and homeostasis. This study further supports the perspective that SIRT6 may be a therapeutic target for IBD.
